# Factors associated with measures of sarcopenia in pre and postmenopausal women

**DOI:** 10.1186/s12905-020-01153-9

**Published:** 2021-01-02

**Authors:** Nirmala Rathnayake, Gayani Alwis, Janaka Lenora, Sarath Lekamwasam

**Affiliations:** 1 Department of Nursing, Faculty of Allied Health Sciences, University of Ruhuna, Galle, Sri Lanka; 2 Department of Anatomy, Faculty of Medicine, University of Ruhuna, Galle, Sri Lanka; 3 Department of Physiology, Faculty of Medicine, University of Ruhuna, Galle, Sri Lanka; 4 Population Health Research Centre, Department of Medicine, Faculty of Medicine, University of Ruhuna, Galle, Sri Lanka

**Keywords:** Muscle strength, Physical performance, Postmenopausal women, Premenopausal women, Skeletal muscle mass

## Abstract

**Background:**

Menopause associated low serum estradiol marks varieties of derangements in muscle mass and functions leading to sarcopenia. This cross-sectional study was carried out to examine the factors associated with measures of sarcopenia; skeletal muscle mass (SMM), muscle strength and physical performance (PP) in a group of premenopausal (PrMW) and postmenopausal women (PMW) selected from Sri Lanka.

**Methods:**

Randomly selected 184 PrMW and 166 PMW from Galle district, Sri Lanka were studied. SMM was measured with duel energy X ray absorptiometry and relative appendicular SMM index (RSMI; kg/m^2^) was calculated. Other measurements made include handgrip strength (HGS; kg) and gait speed (GS; m/s), anthropometric indices, consumption of macro and micronutrients, and pattern of physical activities (PA). A serum sample was analyzed for fasting insulin, serum estradiol and vitamin D. Variables which significantly correlated with RSMI, HGS and GS of PrMW and PMW were separately entered into multiple linear regression models to extract the associated factors.

**Results:**

Mean (SD) age of PrMW and PMW were 42.4 (6.0) and 55.8 (3.8) years respectively. In the regression analysis, RSMI in PrMW showed significant associations with body mass index (BMI), HGS, total-body-fat-mass (TBFM) and weight (adjusted R^2^ = 0.85) and in PMW with BMI, weight, TBFM, hip-circumference and fasting insulin (adjusted R^2^ = 0.80). BMI showed the strongest association with RSMI in both PrMW (r = 0.87, R^2^ = 0.76) and in PMW (r = 0.87, R^2^ = 0.76). HGS in PrMW showed significant associations with appendicular SMM (ASMM), total-body-bone-mineral-content, vigorous PA score, age and weight (adjusted R^2^ = 0.33) and in PMW with ASMM and height (adjusted R^2^ = 0.23). ASMM showed the strongest association with HGS in both PrMW (r = 0.44, R^2^ = 0.20) and PMW (r = 0.44, R^2^ = 0.20). GS in PrMW showed significant associations with height, BMI and energy consumption (adjusted R^2^ = 0.13) while in PMW, with carbohydrate consumption and total-body-bone-mineral-density (adjusted R^2^ = 0.09). While in PrMW, height showed the strongest association with GS (r = 0.28, R^2^ = 0.08) in PMW, it was carbohydrate consumption (r = 0.24, R^2^ = 0.06).

**Conclusions:**

Factors that are associated with different measures of sarcopenia are not uniform and vary widely from anthropometry to nutrient intake indicating that these measures are somewhat independent and are governed by different factors.

## Background

Sarcopenia is a syndrome characterized by progressive loss of skeletal muscle mass (SMM) [1] along with loss of muscle strength and physical performance (PP) that creates adverse outcomes such as physical disability, poor quality of life (QOL) and death [2–4]. Low serum estrogen in the postmenopausal period is the main cause of the rapid decline in SMM, muscle strength and PP seen in old age [5].

Relative appendicular SMM index (RSMI—height adjusted appendicular SMM) which is the primary measure of SMM together with loss of muscle strength and/or PP is used to categorize sarcopenia [1, 6]. Muscle strength and PP are evaluated by handgrip strength (HGS) and gait speed (GS), respectively [1].

SMM is a key component of body composition accounting for 30–40% of total body weight and it correlates with physical functions and general health status [7]. SMM in women peaks around the third decade and decreases gradually afterwards before an accelerated decline after the fifth decade [7]. Women begin to lose muscle strength around their fifth and sixth decade of life [7] and experience about 21% reduction of muscle strength between 25–55 years [8]. The annual decline in PPs is around 1–2% after 50 years and reaches 3% after the age of 60 years [9]. The association observed between muscle strength and circulating estrogen suggests that these changes are partly related to hormonal changes seen around menopause [7]. The poor PP in women compared to men suggest that gender-specific factors across life may influence the maximum PP achieved at the end of linear growth and the rate of decline with age [10, 11].

The pathophysiology of sarcopenia is multifaceted and includes many causes. Primary sarcopenia is involved with the age related declining of SMM and muscle function accompanied by lack of sex hormone, apoptosis and mitochondrial dysfunction. Secondary sarcopenia is associated with endocrine, nutrition, disuse and neurodegenerative disorders [1]. Several mechanisms such as protein synthesis, proteolysis, neuromuscular integrity and muscle fat content may be involved in the onset and progression of sarcopenia. In an individual with sarcopenia, several mechanisms may be involved, and relative contributions may vary over time [1].

Indices of sarcopenia such as RSMI, HGS and PP are under the influence of a multitude of factors and substantial overlaps of these factors are seen. While one measure of sarcopenia is linked with many factors, one particular factor can have influence on several indices of sarcopenia. Apart from low estrogen; age, physical disability, physical inactivity, low testosterone, low vitamin D [12–18] and increased fat mass [19–21], are linked with low SMM in women. Further, muscle strength and PPs are also associated with vitamin D, other nutrients, serum estradiol, inflammatory conditions and physical inactivity [7, 18, 22, 23].

Understanding the factors leading to low SMM, muscle strength and PP in women is important to optimize their physical functions and reduce disability. Even though factors that determines SMM, muscle strength and PP are the focus of current research, most studies has been carried out mainly in European and high-income countries involving elderly populations. The findings of these studies cannot be directly applied to women outside those countries, particularly those in South Asian countries where genetic and non-genetic factors are different from Western populations. Thus, this study was designed to examine the factors associated with measures of sarcopenia i.e., SMM, muscle strength and PP in premenopausal women (PrMW) and postmenopausal women (PMW), selected from the Southern part of Sri Lanka.

## Methods

### Study design, setting and subjects

A community-based cross sectional study was carried out in Galle district, Sri Lanka, from June 2015 to January 2017 [24]. Two groups of apparently healthy community-dwelling PrMW (n = 184, aged 30–55 years) and PMW (n = 166, aged 45–60 years) selected using multi-stage cluster sampling were included in the study. Menopausal status was determined based on the classification of Stages of Reproductive Aging Workshop (STRAW) [24] wherein the premenopausal status was defined by regular or irregular menstruation occurring naturally (PrMW) while women who have not menstruated within the previous 12 months were considered as PMW. Women who used thyroxin, corticosteroids, insulin, hormone replacement therapy or hormonal contraceptives were excluded from the study. Those who were pregnant or lactating, and on dedicated dietary programs or supervised exercise programs (following a dietary program under the supervision of a dietician or aiming to achieve a weight or a BMI target and following an exercise program under the instruction of physical instructor or aiming to achieve a weight or a BMI target) and those who had a chronic disease (non-communicable diseases, chronic infections, polycystic ovary syndrome or chronic major organ diseases) were also excluded.

### Data collection and Measurements

Central-type DXA scanner (Hologic Discovery W, Hologic Inc, Bedford, MA, USA) was used to measure the SMM (kg) adhering to the manufacturer’s protocol. The procedure was carried out by the same technician who calibrated the device on each scanning day. Analytical software provided by the DXA manufacturer was used to analyze SMM. ASMM was calculated by the sum of SMM of all four limbs and the RSMI (kg/m^2^) was calculated using the following formula [25]; RSMI = ASMM in kg /height in meters^2^. In addition, total body fat mass (TBFM), total body bone mineral density (TBBMD) and total body bone mineral content (TBBMC) were measured using the same DXA scanner.

HGS (kg) of the dominant side was measured [26] using the Lafayette hand held dynamometer (Lafayette Instrument Co, Sagamore Parkway North, USA). During the test, the subjects were asked to hold the dynamometer with the dominant hand on upright position with the arm at right angles and the elbow by the side of the body [26].

Four (4) meter customary paced walking; the time taken to walk the central four meters of an eight meter course at a usual self-selected pace was measured to detect the GS (m/s). In order to eliminate the effects of acceleration and deceleration, the initial and final two meters were excluded from the calculation.

Both HGS and GS tests were done twice and were observed by a single trained investigator (the principal investigator of the study). Average of two measurements was used for the analyses [26].

Body weight (kg) and height (m) were measured to the nearest 0.1 kg and 0.1 cm respectively with a calibrated Stadiometer (NAGATA, Tainan, Taiwan). Circumferences (cm) of waist (WC) and hip (HC) were measured to the nearest 0.1 cm with a plastic measuring tape. Body mass index (BMI, kg/m^2^) and waist to hip ratio (WHR) were calculated. All anthropometric indices were measured according to the standard protocol [27] by the same investigator who observed the HGS and GS to ensure the consistency of measurements following standard guidelines.

The pattern of physical activity (PA) was determined with the short version of the international PA questionnaire (IPAQ) [28]. Daily total energy, carbohydrate, protein, fats and calcium consumption were obtained from a 24 h dietary recall (HDR) method. The subjects were asked to recall all foods and beverages, consumed over the previous 24-h period. Respondents were probed for the types of foods and food preparation methods. For uncommon mixed meals, the details of recipes and preparation methods were collected at the time of taking the 24 HDR. All foods recorded in 24 HDR were converted into grams and then, the intake of total energy were analyzed using Indian food composition tables [29] and Sri Lankan food composition tables [30].

A sample of venous blood (4 mL) was drawn from the antecubital vein in the non-dominant side in the morning after the subject had fasted for 10–12 h. Fasting insulin, serum estradiol and vitamin D (25-hydroxyvitamin D (25(OH) D) levels were measured with Enzyme Linked Immuno Sorbent Assay (ELISA) technique. All the investigations were performed in duplicate tests at the standard laboratory premises of the Nuclear Medicine Unit, Faculty of Medicine, University of Ruhuna under expert scientific involvement.

### Statistical analyses

Descriptive statistics; means (SD) or frequency (%), were used to describe the data. Differences between PrMW and PMW were compared using independent sample t test. Correlation between variables and the RSMI, HGS and GS were evaluated with Pearson correlation (r). The variables which showed significant correlations were entered into a multiple regression model in both “enter” and “stepwise” manner to detect significant factors associated with RSMI, HGS and GS. Correlations and regression analyses were done for PrMW and PMW, seperately. The collinearity between variables were verified by the variance inflation factor (VIF) and tolerance (T) values. Thus, VIF values < 10 and T values above 0.1 were considered as acceptable. Data were analysed using SPSS 20.0 and *p* value < 0.05 was considered statistically significant.

### Ethical consideration

Ethical clearance for this study was obtained from the Ethical Review Committee, Faculty of Medicine, University of Ruhuna, Sri Lanka (Reference number; 24.09.2014:3.2). Written informed consent was obtained from each subject before participation.

## Results

### Basic characteristics of PrMW and PMW

Sociodemographic characteristics of the PrMW and PMW who participated in the study were published in our previous work [24]. Mean (SD) age of PrMW and PMW were 42.4 (6.0) and 55.8 (3.8) years respectively. The basic characteristics of study subjects are shown in Table 1. Three main measures of sarcopenia; RSMI, HGS and GS were higher in PrMW compared to PMW (p < 0.01). Variables correlated with RSMI, HGS and PP of PrMW and PMW are shown in Tables 2, 3 and 4 respectively.

**Table 1:** Basic characteristics of PrMW and PMW

Characteristics	PrMW (n = 184) Mean (SD)	PMW (n = 166) Mean (SD)	*p* value *
Measures of sarcopenia			
RSMI (kg/m^2^)	6.9 (0.9)	6.6 (1.0)	0.008
HGS (kg)	19.0 (6.0)	15.2 (4.8)	< 0.001
GS (m/s)	1.2 (0.1)	1.0 (0.16)	< 0.001
Anthropometry indices			
Weight (kg)	58.0 (9.8)	57.0 (11.9)	0.44
Height (m)	1.5 (0.1)	1.4 (0.1)	< 0.001
WC (cm)	82.5 (9.8)	83.3 (12.3)	0.50
HC (cm)	97.0 (8.5)	98.7 (10.1)	0.09
WHR	0.8 (0.1)	0.8 (0.1)	0.47
BMI (kg/m^2^)	24.9 (4.0)	25.9 (4.5)	0.37
Body composition indices			
TBBMD (g/cm^2^)	0.892 (0.060)	0.812 (0.081)	< 0.001
TBBMC (g)	1283.6 (183.1)	1092.8 (250.2)	< 0.001
TBFM (kg)	18.4 (5.6)	19.6 (6.4)	0.30
ASMM (kg)	16.0 (2.5)	14.8 (2.9)	< 0.001
Serum markers			
Vitamin25(OH)D (n/mol)	18.7 (2.1)	18.5 (2.7)	0.34
Estradiol (mlU/L)	149.5 (107.6)	57.3 (55.4)	< 0.001
Fasting insulin (g/dl)	9.9 (5.1)	11.1 (6.6)	0.05
Lifestyle factors			
Physical activity pattern			
Walking PA score (MET/min/week)	829.2 (186.7)	580.2 (156.8)	0.01
Moderate PA score (MET/min/week)	4868.0 (574.2)	4770.12 (857.0)	0.20
Vigorous PA score (MET/min/week)	1785.2 (1784.9)	2297.59 (1917.4)	0.01
Total PA score (MET/min/week)	7482.5 (2400.0)	7648.03 (2534.6)	0.53
Macro and micro nutrients consumption			
Total energy (kcal)	1368.2 (412.5)	1154.1 (331.8)	< 0.001
Protein (g)	38.7 (13.4)	32.2 (12.1)	< 0.001
Fat (g)	30.1 (19.0)	28.1 (19.6)	0.34
Carbohydrates (g)	236.0 (79.2)	195.1 (68.9)	< 0.001
Calcium (mg)	293.8 (180.4)	288.0 (245.3)	0.79

*PrMW* premenopausal women, *PMW* postmenopausal women, *WC* waist circumference, *HC* hip circumference, *WHR* waist to hip ratio, *BMI* body mass index, *ASMM* appendicular skeletal muscle mass, *RSMI* relative ASMM, *HGS* hand grip strength, *GS* gait speed, *TBBMD* total body bone mineral density, *TBBMC* total body bone mineral content, *TBFM* total body fat mass, *PA* physical activities

*p values derived from independent sample t test

**Table 2:** Variables correlated (Pearson’s correlations) with RSMI in PrMW and PMW

PrMW (n = 184)		PMW (n = 166)	
Correlated variables	Correlation (r)	Correlated variables	Correlation (r)
Weight	0.83**	Weight	0.84**
WC	0.80**	WC	0.79**
HC	0.77**	HC	0.79**
BMI	0.87**	BMI	0.87**
WHR	0.47**	WHR	0.26**
TBFM	0.74**	TBFM	0.70**
TBBMD	0.32**	TBBMD	0.46**
TBBMC	0.40**	TBBMC	0.55**
HGS	0.27**	HGS	0.33**
Fasting insulin	− 0.39**	Fasting insulin	− 0.37**
–	–	Vitamin 25(OH)D	0.20**
–	–	Walking PA	0.16*

*PrMW* premenopausal women, *PMW* postmenopausal women, *WC* waist circumference, *HC* hip circumference, *WHR* waist to hip ratio, *BMI* body mass index, *ASMM* appendicular skeletal muscle mass, *RSMI* relative ASMM, *HGS* hand grip strength, *GS* gait speed, *TBBMD* total body bone mineral density, *TBBMC* total body bone mineral content, *TBFM* total body fat mass, *PA* physical activities

**Correlation is significant at the 0.01 level (2-tailed)

*Correlation is significant at the 0.05 level (2-tailed)

**Table 3:** Variables correlated (Pearson’s correlations) with HGS in PrMW and PMW

PrMW (n = 184)		PMW (n = 166)	
Correlated variables	Correlation (r)	Correlated variables	Correlation (r)
Age	− 0.20**	Weight	0.40**
Weight	0.26**	Height	0.40**
Height	0.41**	WC	0.28**
WC	0.19**	HC	0.33**
HC	0.16*	BMI	0.27**
WHR	0.15*	TBFM	0.35**
TBBMD	0.32**	TBBMD	0.37**
TBBMC	0.42**	TBBMC	0.43**
ASMM	0.44**	ASMM	0.44**
GS	0.22**	Serum Estradiol	0.16*
Fasting insulin	− 0.10*	Energy consumption	0.17*
Energy consumption	0.17*	Protein consumption	0.19*
Carbohydrate consumption	0.15*	–	–
Vigorous PA	0.20*	–	–

*PrMW* premenopausal women, *PMW* postmenopausal women, *WC* waist circumference, *HC* hip circumference, *WHR* waist to hip ratio, *BMI* body mass index, *ASMM* appendicular skeletal muscle mass, *RSMI* relative ASMM, *HGS* hand grip strength, *GS* gait speed, *TBBMD* total body bone mineral density, *TBBMC* total body bone mineral content, *TBFM* total body fat mass, *PA* physical activities

**Correlation is significant at the 0.01 level (2-tailed)

*Correlation is significant at the 0.05 level (2-tailed)

**Table 4:** Variables correlated (Pearson’s correlations) with GS in PrMW and PMW

PrMW (n = 184)		PMW (n = 166)	
Correlated variables	Correlation (r)	Correlated variables	Correlation (r)
Height	0.28**	TBBMD	0.18*
BMI	− 0.20**	TBBMC	0.17*
HGS	0.22**	Energy consumption	0.24**
Energy consumption	0.18*	Carbohydrate consumption	0.24**
Carbohydrate consumption	0.16*	–	–

*PrMW* premenopausal women, *PMW* postmenopausal women, *BMI* body mass index, *HGS* hand grip strength, *TBBMD* total body bone mineral density, *TBBMC* total body bone mineral content

**Correlation is significant at the 0.01 level (2-tailed)

*Correlation is significant at the 0.05 level (2-tailed)

### Factors associated with RSMI, HGS and GS in PrMW and PMW

BMI, HGS, TBFM and weight showed significant associations with RSMI in PrMW (R = 0.92, R^2^ = 0.85) while BMI, weight, TBFM, HC and fasting insulin were associated with RSMI among PMW (R = 0.90, R^2^ = 0.80) (Table 5). Among all, BMI showed the strongest association with RSMI in both PrMW (r = 0.87, R^2^ = 0.76) and PMW (r = 0.87, R^2^ = 0.76).

**Table 5:** Factors associated with RSMI of PrMW and PMW in multiple regression analysis

Factors	Unstandardized coefficients		Standardized coefficients	t	Sig
	**B**	**SE**	β		
PrMW (n = 184)					
BMI	0.24	0.01	1.04	13.49	< 0.001
HGS	0.01	0.005	0.10	3.30	0.001
TBFM	0.12	0.01	0.74	8.54	< 0.001
Weight	0.05	0.008	0.55	6.47	< 0.001
Overall model R = 0.92, Adjusted R^2^ = 0.85, SEE = 0.36, Durbin–Watson = 1.81 ANOVA; F = 267.24, *p* < 0.001					
PMW (n = 166)					
BMI	0.18	0.02	0.76	8.05	< 0.001
Weight	0.05	0.01	0.60	5.33	< 0.001
TBFM	0.05	0.01	0.30	3.84	< 0.001
HC	0.02	0.01	0.22	3.28	0.04
Fasting insulin	0.01	0.006	0.07	2.05	0.04
Overall model R = 0.90, Adjusted R^2^ = 0.80, SEE = 0.47, Durbin–Watson = 1.80 ANOVA; F = 137.79, *p* < 0.001					

*PrMW* premenopausal women, *PMW* postmenopausal women, *BMI* body mass index, *HGS* hand grip strength, *TBFM* total body fat mass

ASMM, TBBMC, vigorous PA score, age and weight showed significant associations with HGS in PrMW (R = 0.58, R^2^ = 0.33) while ASMM and height showed significant associations with HGS in PMW (R = 0.48, R^2^ = 0.23) (Table 6). ASMM showed the strongest association with HGS in both PrMW (r = 0.44, R^2^ = 0.19) and PMW (r = 0.44, R^2^ = 0.19).

**Table 6:** Factors associated with HGS of PrMW and PMW in Multiple Regression Analysis

Factors	Unstandardized Coefficients		Standardized Coefficients	t	Sig
	B	SE	β		
PrMW (n = 184)					
TBBMC	0.11	0.003	0.31	3.85	< 0.001
Age	− 0.19	0.06	− 0.19	− 3.21	0.002
Vigorous PA	0.00	0.00	0.12	1.97	0.04
ASMM	1.37	0.34	0.57	4.04	< 0.001
Weight	0.26	0.08	0.42	3.19	0.002
Overall model R = 0.58, Adjusted R^2^ = 0.33, SEE = 5.06, Durbin–Watson = 1.76 ANOVA; F = 18.02, *p* < 0.001					
PMW (n = 166)					
ASMM	0.53	0.14	0.31	3.80	< 0.001
Height	17.17	6.41	0.24	2.67	0.008
Overall model R = 0.48, Adjusted R^2^ = 0.23, SEE = 4.28, Durbin–Watson = 1.90 ANOVA; F = 24.86, *p* < 0.001					

*PrMW* premenopausal women, *PMW* postmenopausal women, *ASMM* appendicular skeletal muscle mass, *TBBMC* total body bone mineral content, *PA* physical activities

GS in PrMW showed significant associations with height, BMI and energy consumption (R = 0.37, R^2^ = 0.13) while in PMW, carbohydrate consumption and TBBMD showed significant associations with GS (R = 0.30, R^2^ = 0.09) (Table 7). The strongest factor associated with GS in PrMW was height (r = 0.28, R^2^ = 0.08) while in PMW, it was carbohydrate consumption (r = 0.24, R^2^ = 0.06).

**Table 7:** Factors associated with GS of PrMW and PMW in Multiple Regression Analysis

Factors	Unstandardized Coefficients		Standardized Coefficients	t	Sig
	B	SE	β		
PrMW (n = 184)					
Height	0.75	0.21	0.25	3.58	< 0.001
BMI	− 0.008	0.003	− 0.17	− 2.53	0.01
Energy consumption	6.41	< 0.001	0.15	2.18	0.03
Overall model R = 0.37, Adjusted R^2^ = 0.13, SEE = 0.16, Durbin–Watson = 1.68 ANOVA; F = 9.50, *p* < 0.001					
PMW (n = 166)					
Carbohydrate consumption	0.001	0.00	0.24	3.26	0.001
TBBMD	0.35	0.14	0.17	2.34	0.02
Overall model R = 0.30, Adjusted R^2^ = 0.09, SEE = 0.15, Durbin–Watson = 1.60 ANOVA; F = 8.52, *p* < 0.001					

*PrMW* premenopausal women, *PMW* postmenopausal women, *BMI* body mass index, *TBBMD* total body bone mineral density

## Discussion

This study revealed that a diversity of factors including measures of anthropometry, body composition and lifestyle, is associated with the three main measures of sarcopenia in PrMW and PMW. Though studies on factors associated with muscle mass in women are common, studies focusing on all three measures of sarcopenia in a single study are scarce.

Similar to our findings, previous studies have shown that anthropometric indices [14, 20, 31, 32] and TBFM [19] are closely associated with SMM in women. Similarly, Maltais et al. [7] have shown fasting insulin to be associated with SMM in PMW. SMM is the major metabolically active body compartment for the disposal of glucose in healthy individuals [33]; therefore, the loss of SMM can disrupt this mechanism resulting in hyperinsulinemia. Even though previous studies have reported SMM in women to be associated with low PA [19, 34, 35], low protein intake [36–39] and hypovitaminosis D [7, 17, 18, 40], these factors showed only minor correlations with RSMI in the current study. The step-wise regression model excluded them as weak associations. These inconsistencies may be due to the differences in the selection of subjects and the way measurements were taken. Furthermore, the differences could be due to the variations in lifestyle including diet, degree of PA, and economy between communities.

Concordant with our observations, HGS has been shown to be associated with ASMM [41–43] in women. Although there is no linear relationship between muscle mass and muscle function, ASMM is a strong predictor of HGS. Da Camara et al. found women with higher BMI to have higher HGS [44] and this is similar to the association we found between HGS and height and weight. This could be due to the fact that women with higher BMI could possess higher SMM; hence likely to have greater HGS. Keeping with our observation, low PA is linked with low HGS [2, 45, 46] in women. It is possible that PA enhance muscle strength by stimulating myofibrillar muscle protein synthesis and inhibiting muscle protein breakdown [47]. Though we found a significant association between bone mineral content (BMC) and HGS in PrMW, previous studies showing such association is scarce. A significant association between osteoporosis and muscle strength, however, has been observed [48] in PMW, previously. This could be due to the fact that the risk factors of osteoporosis and sarcopenia, including age, genetics, endocrine function, and mechanical factors are similar [49–51].

Keeping in line with our observations, GS has shown significant associations with low height [52], higher BMI [53] and low bone mineral density (BMD) [54]. The associations seen between GS and height and BMI are understandable. The gait step length is possibly influenced by height while subjects with higher BMI may have poor mobility and lower GS. Association between BMD and GS is understandable as higher GS would mean higher PA and in turn, higher bone density and mineral content [55]. Though our study observed both energy and carbohydrate consumption are linked with GS in both PrMW and PMW, we were unable to find previous studies supporting these associations.

The positive associations seen between measures of sarcopenia and nutritional factors, anthropometric indices, and body composition indices have the potential to be utilized in future health promotion activities. Women in Sri Lanka tend to neglect their nutritional requirements amidst many family responsibilities. They also tend to change from non-vegetarian to vegetarian diet due to religious influences especially in the postmenopausal period. Young women are likely to have a sedentary lifestyle which could lead to loss of SMM, functional limitations and derangements of BMD and BMC. Therefore, health education programs need to focus on food patterns of young women to maintain optimal SMM and its functions. Further, a physically active lifestyle should be promoted among both young and old women focusing on aerobics, strength and balance training activities.

Apart from health promotion at the community level, this information would help clinicians in patient care. Although sarcopenia is not the primary reason to seek medical care, it may co-exist in patients presenting with emphysema, heart failure, falls, fractures, frailty and diabetes. Furthermore, acute sarcopenia may exacerbate these conditions and influence the clinical outcome. Clinicians need to be aware of this possibility and the improvement of muscle mass and functions should be an integral part of the management of such patients. Further, the variation of factors that is associated with the components of sarcopenia between PrMW and PMW need to be clarified.

The current study is a cross-sectional study involving a single geographical area, which limits the generalizability of findings. However, we evaluated many factors that are interconnected with the measures of sarcopenia and this is the first detailed investigation on factors linked with sarcopenic measures among Sri Lankan women. Therefore the findings would lay the foundation for future research.

## Conclusions

We found that many factors are associated with the measures of sarcopenia. BMI was the most significant factor associated with RSMI while ASMM was the most significant factor for HGS of both PrMW and PMW. GS was mostly associated with height and carbohydrate consumption in PrMW and PMW, respectively. The findings suggest that the factors associated with the measures of sarcopenia are not uniform and vary widely from simple body measurements such as anthropometry to nutrient intake irrespective of menopausal status. It emphasizes that these measures of sarcopenia are somewhat independent and are governed by different factors.

## Data Availability

The datasets used and/or analyzed during the current study are available from the corresponding author on reasonable request.

## Abbreviations

SMM: Skeletal muscle mass
PP: Physical performance
PrMW: Premenopausal women
PMW: Postmenopausal women
DXA: Dual energy x ray absorptiometry.
RSMI: Relative appendicular skeletal muscle mass index.
ASMM: Appendicular skeletal muscle mass.
HGS: Hand grip strength.
GS: Gait speed.
PA: Physical activities.
SD: Standard deviation.
BMI: Body mass index.
TBFM: Total body fat mass.
TBMBC: Total body bone mineral content.
TBBMD: Total body bone mineral density.
QOL: Quality of life.
WC: Waist circumference.
HC: Hip-circumference.
WHR: Waist to hip ratio.
IPAQ: International PA questionnaire.
HDR: 24 H dietary recall.
ELISA: Enzyme Linked Immuno Sorbent Assay.
VIF: Variance inflation factor.
T: Tolerance.
STRAW: Stages of Reproductive Aging Workshop.
BMD: Bone mineral density.
BMC: Bone mineral content
